# Dynamic Variations in Infrared Skin Temperature of Weaned Pigs Experimentally Inoculated with the African Swine Fever Virus: A Pilot Study

**DOI:** 10.3390/vetsci8100223

**Published:** 2021-10-09

**Authors:** Sang-Ik Oh, Hu Suk Lee, Vuong Nghia Bui, Duy Tung Dao, Ngoc Anh Bui, Thanh Duy Le, Minh Anh Kieu, Quang Huy Nguyen, Long Hoang Tran, Kyoung-Min So, Seung-Won Yi, Eunju Kim, Tai-Young Hur

**Affiliations:** 1 Division of Animal Diseases & Health, National Institute of Animal Science, Rural Development Administration, 1500 Kongjwipatjwi-ro, Wanju 55365, Korea; ls2273@korea.kr (K.-M.S.); swfs0619@korea.kr (S.-W.Y.); keunjunim@korea.kr (E.K.); tyohur@korea.kr (T.-Y.H.); 2 International Livestock Research Institute (ILRI), Hanoi, Vietnam; H.S.Lee@cgiar.org (H.S.L.); Nqhuy288@gmail.com (Q.H.N.); 3 Virology Department, National Institute of Veterinary Research, Hanoi, Vietnam; buinghiavuong@gmail.com (V.N.B.); ddtung83@yahoo.com (D.T.D.); buingocanh_1980@yahoo.com (N.A.B.); Lethanhduytb74@gmail.com (T.D.L.); kieuanhminh94@gmail.com (M.A.K.); longhoang9998@gmail.com (L.H.T.)

**Keywords:** African swine fever, infrared thermography, infrared skin temperature, fever, pig

## Abstract

African swine fever (ASF) is a devastating viral disease in pigs and is therefore economically important for the swine industry. ASF is characterized by a short incubation period and immediate death, making the early identification of ASF-infected pigs essential. This pilot-scale study evaluates whether the infrared thermography (IRT) technique can be used as a diagnostic tool to detect changes in skin temperature (*T_sk_*) during the early stages of disease development in experimentally ASF-infected pigs. Clinical symptoms and rectal temperatures (*T_core_*) were recorded daily, and IRT readings during the experimental ASF infection were analyzed. All infected pigs died at 5–8 days post inoculation (dpi), and the incubation period was approximately 4 dpi. The average *T_core_* increased from 0 dpi (38.9 ± 0.3 °C) to 7 dpi (41.0 ± 0.5 °C) and decreased by 8 dpi (39.8 ± 0 °C). The maximum *T_sk_* of ASF-infected pigs increased from 2 (35.0 °C) to 3 dpi (38.5 °C). The mean maximum *T_sk_* observed from three regions on the skin (ear, inguinal, and neck) significantly increased from 2 to 3 dpi. This study presents a non-contact method for the early detection of ASF in infected pigs using thermal imaging at 3 days after ASF infection.

## 1. Introduction

Fever or internal temperature (*T_core_*) in pigs is commonly assessed by measuring rectal temperature with a mercurial thermometer. However, this method is invasive and requires manual handling of animals, which might increase the risk of spreading infectious pathogens within farms [1,2]. Infrared thermography (IRT) is a technique that creates a visible image from the invisible infrared radiation emitted by an object [3]. This technique has been increasingly used to detect several illnesses in humans and animals. Recently, IRT-based remote screening technology was widely used as a tool to measure temperature in the ongoing COVID-19 pandemic in humans [3]. In pigs, IRT has been used for the early detection of infections caused by *Actinobacillus pleuropneumoniae*, *Salmonella* Typhimurium, and *Escherichia coli* [4,5,6]. It is a useful tool for detecting fever without manual handling of individual pigs and, thus, could reduce the risk of spreading the infection between or within the farms. Therefore, visual surveillance by infrared skin temperature (*T_sk_*) measurement has a great advantage in the early detection of infectious diseases that are accompanied by fever.

African swine fever (ASF) is a fatal disease causing serious economic losses in the pig industry [7]. Previous studies revealed that the incubation period of the African swine fever virus (ASFV) was approximately 3–5 days, and infected pigs died within 7–13 days post inoculation (dpi) [8,9,10,11]. Due to the short incubation period and immediate death, ASFV gives a short window for the disease to be reported by pig farms [8]. Moreover, the main control strategies during ASF outbreaks are livestock culling and quarantine, as there is no vaccine available. Since high fever is one of the most important clinical signs of ASF infection [12], the *T_sk_* measurement method could be practically applied for surveillance of ASF in endemic/affected areas [1].

Pig hair is much coarser than other mammal hair; therefore, it has been suggested that the *T_sk_* of pigs might not reflect the *T_core_* [13]. In order to utilize IRT as a disease diagnostic tool, it is necessary to evaluate the correlation between *T_sk_* and *T_core_* of the infected pigs. However, most studies on IRT have been limited to healthy pigs [1,13,14,15], and only a few studies have addressed the *T_sk_* in the case of porcine respiratory disease [5,6]. Therefore, this study investigates the dynamic comparison between *T_sk_* recorded from thermal infrared (TIR) images of the ASFV-infected pig herd and their *T_core_*. We also analyzed the TIR images of ASFV-infected pigs from three regions of interest (ROI), including ear, inguinal, and neck regions. This pilot study demonstrates the use of IRT for the identification of ASFV-infected pigs in the early stage of infection, paving the way for developing a diagnostic tool for automatic fever detection in the future.

## 2. Materials and Methods

### 2.1. Animals

This preliminary study was part of a larger research project that involved an ASFV challenge experiment to evaluate the pathogenicity of ASFV isolated in Vietnam [9]. A total of 10 pathogen-free pigs (Yorkshire × Landrace × Duroc) aged appropriately 50 days, originating from the same herd in a commercial pig farm, were used. The study was performed in the animal biosecurity level 2 enhanced facility at the National Institute of Veterinary Research, Hanoi, Vietnam. All healthy pigs were moved into the facility one week before the start of the experiment. All pigs were tested and found to be negative for five porcine viral pathogens—namely, foot-and-mouth disease virus, porcine circovirus 2, porcine respiratory and reproductive syndrome virus, classic swine fever virus, and ASFV. Pigs were managed and fed twice a day by the veterinary staff, and water was provided ad libitum. Standard cleaning and maintenance procedures for the pig room were followed. The room temperature and humidity at the biosafety facility during the experimental period are given in Table 1.

All animal procedures followed the guidelines approved by the Animal Ethics Committee of the National Institute of Animal Science in the Republic of Korea and the rules of the National Institute of Veterinary Research in Vietnam. The study was approved by the Institutional Animal Care and Use Committee of the National Institute of Animal Science in the Republic of Korea (Approval Number: NIAS 2020-463).

### 2.2. Experimental Design

**Figure 1:**
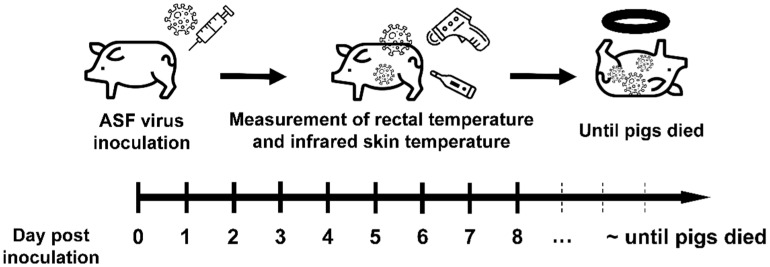
Experimental scheme for the study. The pigs (*n* = 10) were inoculated with ASF virus strain (10^3.5^ HAD_50_/mL) from Vietnam, and the rectal and infrared skin temperatures were measured for seven pigs once a day until the pigs died.

The detailed scheme for the ASFV inoculation experiment is shown in Figure 1. After a 7-day acclimatization period, 10 pigs were inoculated intramuscularly with ASFV, with a dose of 10^3.5^ 50% haemadsorbing doses per mL (HAD_50_/mL). The dose was selected based on an earlier report about efficient induction of infection via the intramuscular route in the case of ASFV from China [16]. The ASFV strain used in this study was VNUA/HY/Vietnam (Genotype II, GenBank accession no. MK554698), which had a 10-nucleotide insertion (5′-GGAATATATA-3′) [17,18]. Primary porcine alveolar macrophages in Dulbecco’s modified Eagle medium (supplemented with 5% fetal bovine serum) were used for culturing the ASFV strain. The clinical symptoms observed after inoculation were previously described in our prior study [9] and were generated based on the protocol by Galindo-Cardiel et al. [19], with minor modifications. The IRT measurement was continued with 7 pigs out of 10 pigs, as clear TIR images could not be obtained from 3 pigs. The *T_core_* of 7 ASFV-infected pigs was recorded daily by the designated mercurial thermometers for each pig. Blood samples were collected every day for quantifying the ASFV infection by qPCR. TIR images of ASFV-infected pigs were obtained by a handheld portable imaging camera (PTi120, Fluke Corp., Everett, WA, USA). The thermal camera used in this study had a spectral range of 8–14 μm, an accuracy of ±2 °C, a spatial resolution of 7.6 mrad, and thermal sensitivity of 60 mk at 30 °C. The TIR image of the ASFV-infected herd was recorded daily in the morning with the camera positioned at a distance of 50–100 cm from the herd. This was then followed by recording the images from individual ASF-infected pigs at dorsal and ventral views. From these images, we analyzed the *T_sk_* of three ROI, including ear, inguinal, and neck.

### 2.3. Data Analysis

The time-series TIR images of ASFV-infected pigs were analyzed using Fluke Connect SmartView Software (Fluke Corp.) to deduce the maximum surface temperature of the selected areas. The software was set to treat each image in the color palette according to the skin temperature in the range of 26–41 °C, with an emissivity of 0.98. The maximum skin temperature was recorded and analyzed every day from the TIR image of the herd as well as the TIR images (from three ROI) of individual pigs.

### 2.4. Statistical Analysis

Statistical analyses were performed using SPSS version 25.0 (IBM, Armonk, NY, USA). A paired *t*-test was conducted to compare the mean values of temperatures between two time points. Results with *p* < 0.05 were considered significant. Temperature data are presented as the mean ± standard deviation calculated based on daily measurements in individual pigs.

## 3. Results

### 3.1. Clinical Assessment of ASF-Infected Pigs

During the period of the experiment, ASFV-infected pigs started to die at 5 days post inoculation (dpi) until 8 dpi. The main clinical signs of ASFV infection were observed from 4 dpi in all of the tested pigs, including digestive and respiratory problems. The mean incubation period was calculated as 3.7 ± 0.5 dpi, as described in our previous study [9]. The pigs died before reaching the predetermined humane endpoint of the study despite close monitoring.

### 3.2. T_core_ of ASF-Infected Pigs

The average *T_core_* of ASF-infected pigs gradually increased as follows: 38.9 ± 0.3 (0 dpi, *n* = 7), 39.2 ± 0.2 (1 dpi, *n* = 7), 38.7 ± 0.3 (2 dpi, *n* = 7), 40.1 ± 0.4 (3 dpi, *n* = 7), 40.9 ± 0.2 (4 dpi, *n* = 7), 40.5 ± 0.5 (5 dpi, *n* = 6), 41.4 ± 0.2 (6 dpi, *n* = 4), 41.0 ± 0.5 (7 dpi, *n* = 4), and 39.8 ± 0 °C (8 dpi, *n* = 1). A significant difference was observed between 1–2 dpi (*p* = 0.019), 2–3 dpi (*p* < 0.001), and 3–4 dpi (*p* = 0014). The maximum *T_core_* of an alive ASF-infected pig also increased as follows: 39.5 (0 dpi), 39.6 (1 dpi), 39.2 (2 dpi), 40.7 (3 dpi), 41.5 (4 dpi), 41.2 (5 dpi), 41.6 (6 dpi), 41.7 (7 dpi), and 41.5 °C (8 dpi).

### 3.3. T_sk_ of ASF-Infected Pigs

**Figure 2:**
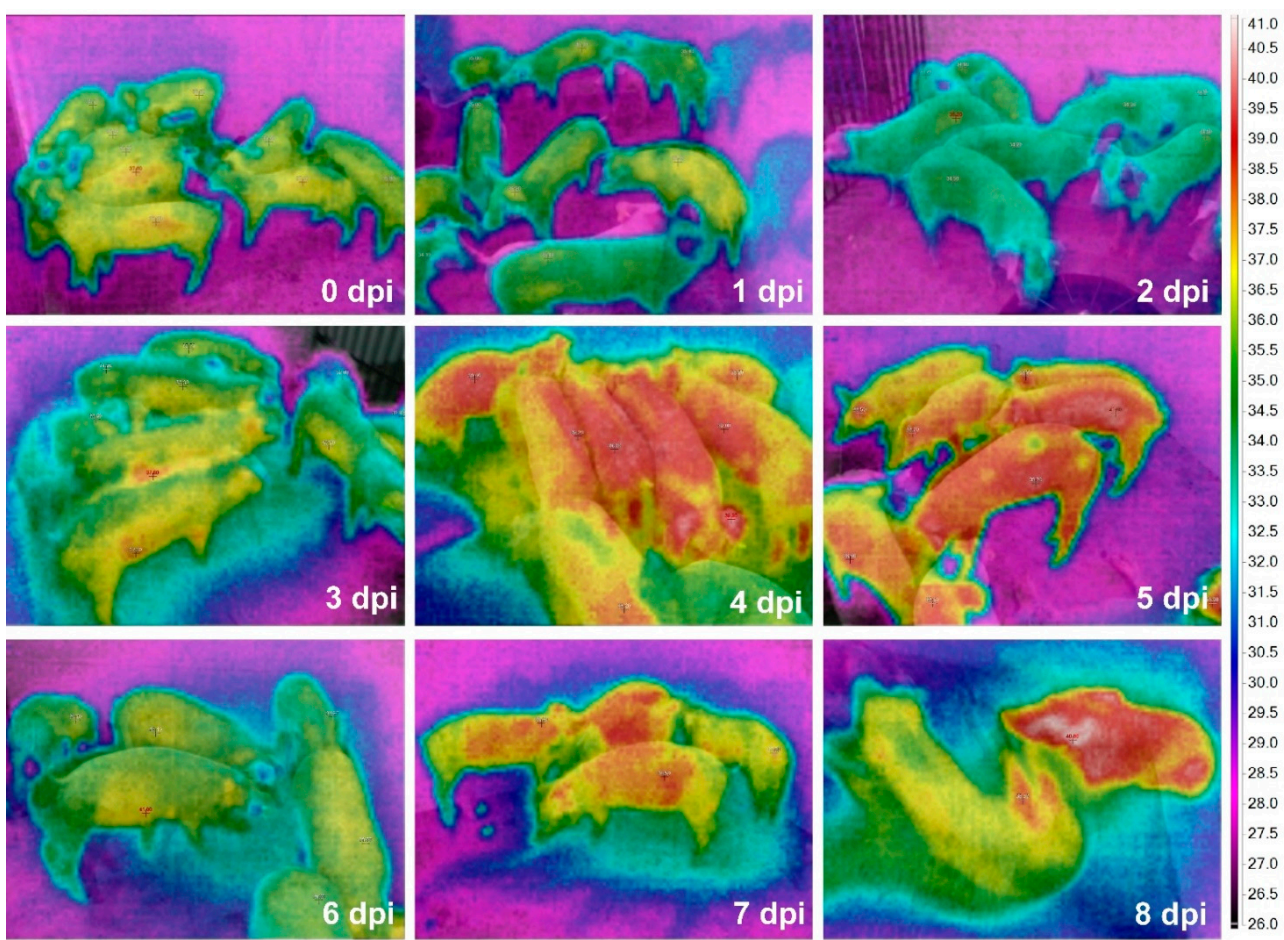
Time-serial thermal images of the pig herd including 50-day-old weaned pigs after ASF virus inoculation.

**Figure 3:**
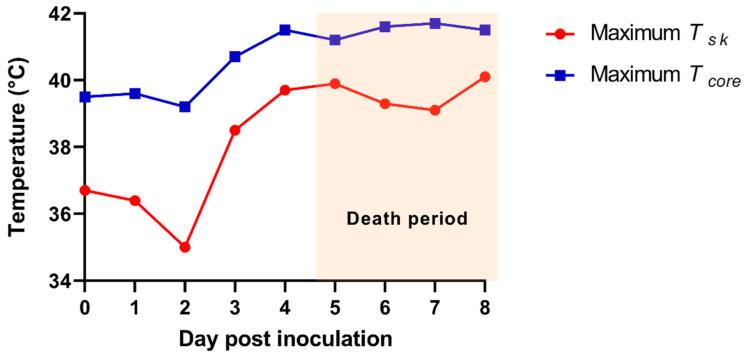
The maximum skin infrared temperature (*T_sk_*) from the thermal infrared images of the African-swine-fever-virus-infected herd (red line). The maximum rectal temperature (*T_core_*) of the pig from the African-swine-fever-virus-infected herd (blue line).

The time-serial TIR images from the ASF-infected herd are shown in Figure 2. The maximum *T_sk_* of an individual pig among the alive ASF-infected pigs were 36.7 (0 dpi), 36.4 (1 dpi), 35.0 (2 dpi), 38.5 (3 dpi), 39.7 (4 dpi), 39.9 (5 dpi), 39.3 (6 dpi), 39.1 (7 dpi), and 40.1 °C (at 8 dpi), representing a greater increase of the maximum *T_sk_* from 2 (35.0 °C) to 3 dpi (38.5 °C). The comparison data between maximum *T_core_* and *T_sk_* are shown in Figure 3.

### 3.4. T_sk_ of ROI from ASF-Infected Pigs

**Figure 4:**
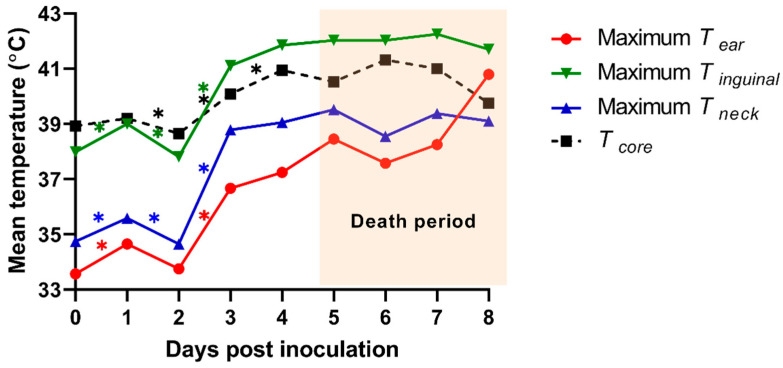
Mean of the maximum infrared skin temperatures of the three regions of interest [the ear (*T_ear_*), inguinal (*T_inguinal_*), and neck (*T_neck_*) region] and the mean of rectal temperature (*T_core_*) from experimentally ASF-infected pigs. * *p* < 0.05.

*T_sk_* of the 3 ROI (ear, inguinal, and neck regions) from an individual pig was analyzed using the TIR images of the dorsal and ventral view. We calculated the mean temperature from maximum *T_sk_* of 3 ROIs and compared them with those from *T_core_* (Figure 4). The means of the maximum ear skin temperature (*T_ear_*) in each pig were 33.6 ± 0.3 (0 dpi), 34.7 ± 0.6 (1 dpi), 33.8 ± 1.1 (2 dpi), 36.7 ± 0.5 (3 dpi), 37.2 ± 1.7 (4 dpi), 38.5 ± 0.7 (5 dpi), 37.6 ± 0.8 (6 dpi), 38.3 ± 1.7 (7 dpi), and 40.8 ± 0 °C (8 dpi). The means of the maximum inguinal temperature (*T_inguinal_*) were 38.0 ± 0.5 (0 dpi), 39.0 ± 0.3 (1 dpi), 37.8 ± 0.9 (2 dpi), 41.1 ± 0.9 (3 dpi), 41.9 ± 0.6 (4 dpi), 42.0 ± 0.3 (5 dpi), 42.0 ± 0.7 (6 dpi), 42.3 ± 0.6 (7 dpi), and 41.7 ± 0 °C (8 dpi). The means of the maximum neck skin temperature (*T_neck_*) were 34.7 ± 0.5 (0 dpi), 35.6 ± 0.7 (1 dpi), 34.7 ± 0.8 (2 dpi), 38.8 ± 0.8 (3 dpi), 39.1 ± 0.4 (4 dpi), 39.5 ± 0.5 (5 dpi), 38.6 ± 0.5 (6 dpi), 39.4 ± 0.8 (7 dpi), and 39.1 ± 0 °C (8 dpi). The maximum *T_ear_* indicated a significant difference between 1 and 2 dpi (*p* = 0.021) as well as 2 and 3 dpi (*p* < 0.001). We also observed significant differences 0–1 dpi (*p* = 0.001 and 0.004), 1–2 dpi (*p* = 0.044 and 0.027), and 2–3 dpi (*p* < 0.001 and *p* = 0.001) in the maximum *T_inguinal_* and *T_neck_*, respectively.

## 4. Discussion

ASF is a fatal viral disease that causes hemorrhagic fever in domestic pigs and has been included in the list of notifiable diseases by the World Organization for Animal Health (OIE) [20,21]. Given that the ASF outbreaks in the Asia-Pacific region are characterized by a short incubation period and immediate death, the early recognition of ASFV-infected pigs for stamping out from the herd is vital for the successful eradication of ASF [9,16,22,23]. For this reason, our previous study suggested that the rope-based oral fluid sampling technique could be useful for the early detection of ASFV in pigs at the herd level by minimizing contact with pigs [9]. In this study, we investigated the possibility of early detection of ASF-infected pigs by using a thermal camera.

Fifty-day-old weaned pigs experimentally infected by ASFV showed clinical symptoms from 4 dpi and started to die from 5 dpi. In addition, the mean *T_core_* of ASF-infected pigs (*n* = 7) significantly increased from 2 to 4 dpi in this study. These results are consistent with the previous studies that reported the occurrence of clinical symptoms about 4–5 days after ASFV infection [12,16,24,25]. Both the maximum *T_sk_* and *T_core_* from the ASFV-infected herd dramatically increased from 2 to 3 dpi, as shown in Figure 2 and Figure 3. This finding suggested that the real-time daily measurement of *T_sk_* could assist in the early detection for ASFV-infected pigs (3 days after an ASF infection) before they show clinical signs (3.7 ± 0.5 dpi). However, since many other infectious pathogens cause high fever in pigs [4,5,6], it is essential to consider the epidemiological situation prevalent (e.g., ASF outbreak in the nation, neighboring regions, or wild boars) for applying these IRT methods as an ASF diagnostic assist tool. A laboratory analysis will also be required to confirm whether the suspected pigs are infected with ASFV or not.

The results also confirmed that the *T_sk_* from ASFV-infected pigs was distinct from *T_core_*, which was coincident with previous studies [1,13,14,16,26]. Previous studies have also revealed the relationship between *T_sk_* and *T_core_* in pigs, indicating that they differ depending on the age and breed of pigs, ambient temperature, and region of body measured [13,16]. For the effective diagnosis of the ASFV infection in pig farms, it is important to ascertain the optimal *T_sk_* of ASFV-infected pigs. This finding pointed to the possibility that the ASFV-infected weaned pigs (50-day-old) could be identified at an early stage of infection (at least 3 dpi) by analyzing the TIR image of the pig herd. The overall results implied that the weaned pigs with *T_sk_* > 38.5 °C in the TIR image from the herd were considered as potentially infected with ASFV.

Although the TIR images from the herd might help pick pigs with suspected ASFV infection, among the herd, the determination *T_sk_* of the specific areas on the body (ROI) is required to confirm ASFV-infected pigs individually. Previous studies reported that the “thermal windows” of pigs, such as the sulcus auriculae posterior, eye, and vulva, correlated with the *T_core_* [27,28,29]. In this study, we analyzed the *T_sk_* of three ROIs (ear, inguinal, and neck regions) in pigs after ASFV inoculation. The discrepancy of the *T_sk_* among three ROIs was caused by the difference in the blood perfusion to the outer skin surface, the coarseness of pig hair, and the thickness of the fat layer. The results showed that the mean of maximum *T_ear_*, *T_inguinal_*, and *T_neck_* significantly increased from 2 to 3 dpi. However, *T_inguinal_* and *T_neck_* also showed a significant increase at 0–1 dpi, which cannot be otherwise differentiated between normal and ASFV-infected pigs. Moreover, measuring *T_inguinal_* and *T_neck_* requires physical contact of farmworkers with the pigs; hence, these two ROIs present the risk of spreading the virus within farms through the workers handling these pigs. On the other hand, the pig’s ear is an important thermoregulatory area where blood vessels are exposed to the outer skin surface [13]. Several studies have highlighted that the *T_ear_* highly correlates with *T_core_*, as compared with other ROIs [13,26,30]. Notably, our findings showed that the mean of maximum *T_ear_* in ASFV-inoculated pigs dramatically increased (by 2.9 °C) from 2 to 3 dpi, implying the daily measurement of *T_ear_* was useful for detecting ASFV-infected pigs. The overall findings implied that ASFV infection could be suspected in pigs whose *T_ear_* exceeds 36.7 °C.

To the best of our knowledge, this is the first pilot study that investigates the dynamic variation in *T_sk_* of experimentally ASFV-infected pigs. However, the current study has two major limitations. Firstly, only 50-day-old weaned pigs were considered for the experiment. For conclusive inference, the *T_sk_* of pigs from varied age groups needs to be investigated, which can be achieved by using the TIR camera in pig farms. Secondly, the viral inoculation experiment was performed in a biosafety facility where the ambient temperature and humidity were controlled during the experiment. Although the air temperature and humidity were changed from 3 and 4 dpi in this study, we could not clearly identify the impact of these changes on the *T_sk_* of pigs. The *T_sk_* of pigs is known to be influenced by the ambient temperature, and hence, the *T_sk_* of ASFV-infected pigs needs to be monitored under different ambient temperatures [1,26]. Nevertheless, this study provides significant evidence for the potential use of IRT as a method for early detection of ASFV infection in pigs.

## 5. Conclusions

In this study, we suggest the use of IRT for the early detection of ASFV-infected pigs. To summarize, our proposed IRT method for detecting ASFV-infected pigs is described as follows; 1. Daily screening of the herd of pigs using a thermal camera; 2. selecting the pigs with *T_sk_* > 38.5 °C; 3. measuring the maximum *T_ear_*; 4. if the maximum *T_ear_* is more than 36.7 °C or increased more than 2.9 °C within 24 h, the pigs could be suspected to have been infected with ASFV. This IRT method can be used for pig farms that are epidemiologically associated with the high risk of an ASF outbreak. Since the current study was conducted only on pigs in a controlled environment, further research to identify optimal *T_sk_* and *T_ear_* considering varied age groups and ambient temperatures is required for the successful application of the IRT technique to pig farms. Nonetheless, the current findings suggest that IRT can be developed as a diagnostic tool for early detection before the development of clinical symptoms of ASFV infection in pigs.

## 6. Patents

The authors S.-I.O., K.-M.S. and T.-Y.H. declare that they are named inventors on the pending, unpublished patent application in the Republic of Korea (Patent title: “Estimating method and system for recognizing African swine fever infected pigs using the thermal camera”, Patent No. 10-2021-0047517), relating to the use of a thermal camera for early detection of ASF-suspected pigs described herein.

## Figures and Tables

**Table 1:** The temperature and humidity in the biosafety facility during the viral inoculation experiment.

Day Post Inoculation (Days)	0	1	2	3	4	5	6	7	8
Temperature (°C)	28	28	28	28	27	27	27	27	27
Humidity (%)	70	70	70	70	68	65	65	70	69

## Data Availability

The data presented in this study are available by reasonable request from the corresponding author.
